# Effect of local anaesthesia and/or analgesia on pain responses induced by piglet castration

**DOI:** 10.1186/1751-0147-53-34

**Published:** 2011-05-31

**Authors:** Monica Hansson, Nils Lundeheim, Görel Nyman, Gunnar Johansson

**Affiliations:** 1 Department of Animal Breeding and Genetics, Swedish University of Agricultural Scienes, P.O. Box 7023, SE-750 07 Uppsala, Sweden; 2 Department of Animal Environment and Health, Swedish University of Agricultural Scienes, P.O. Box 234, SE-532 23 Skara, Sweden; 3 Swedish Animal Health Service, SE-532 89 Linköping, Sweden

## Abstract

**Background:**

Surgical castration in male piglets is painful and methods that reduce this pain are requested. This study evaluated the effect of local anaesthesia and analgesia on vocal, physiological and behavioural responses during and after castration. A second purpose was to evaluate if herdsmen can effectively administer anaesthesia.

**Methods:**

Four male piglets in each of 141 litters in five herds were randomly assigned to one of four treatments: castration without local anaesthesia or analgesia (C, controls), analgesia (M, meloxicam), local anaesthesia (L, lidocaine), or both local anaesthesia and analgesia (LM). Lidocaine (L, LM) was injected at least three minutes before castration and meloxicam (M, LM) was injected after castration. During castration, vocalisation was measured and resistance movements judged. Behaviour observations were carried out on the castration day and the following day. The day after castration, castration wounds were ranked, ear and skin temperature was measured, and blood samples were collected for analysis of acute phase protein Serum Amyloid A concentration (SAA). Piglets were weighed on the castration day and at three weeks of age. Sickness treatments and mortality were recorded until three weeks of age.

**Results:**

Piglets castrated with lidocaine produced calls with lower intensity (*p *< 0.001) and less resistance movements (*p *< 0.001) during castration. Piglets that were given meloxicam displayed less pain-related behaviour (huddled up, spasms, rump-scratching, stiffness and prostrated) on both the castration day (*p *= 0.06, n.s.) and the following day (*p *= 0.02). Controls had less swollen wounds compared to piglets assigned to treatments M, L and LM (*p *< 0.001). The proportion of piglets with high SAA concentration (over threshold values 200, 400 mg/l) was higher (*p *= 0.005; *p *= 0.05) for C + L compared to M + LM. Ear temperature was higher (*p *< 0.01) for controls compared to L and LM. There were no significant treatment effects for skin temperature, weight gain, sickness treatments or mortality.

**Conclusions:**

The study concludes that lidocaine reduced pain during castration and that meloxicam reduced pain after castration. The study also concludes that the herdsmen were able to administer local anaesthesia effectively.

## Background

Each year approximately 1.5 million male piglets are surgically castrated in Sweden. The number for all EU countries is approximately 100 million. The castration is mainly performed to eliminate boar taint in the meat, but also to prevent aggressive and sexual behaviour of male pigs. Castration is performed within the piglet's first week of life and is traditionally carried out without anaesthesia and analgesia. As surgical castration induces pain in piglets the procedure is considered an important animal welfare issue [1].

Pain is subjective and therefore difficult to quantify, and there are no specific parameters for measuring it [2]. However, it is widely accepted that piglets may react to pain in three ways: trough vocalisation, physiologically, and behaviourally [3]. Although piglets usually vocalise a lot when they are handled there is a clear difference in their vocalisation between being handled and castrated. Piglets that are castrated without anaesthesia produce a higher number of calls and with a higher frequency compared to piglets castrated with anaesthesia [2,4] or sham-castrated piglets (handled identically but without castration) [5-7]. The greatest amount of high-frequency calls are produced when the piglet's spermatic cords are pulled and severed, and is therefore identified as the most painful moment during castration [8].

The sympathetic nervous system is activated during different kinds of stress (pain, anger and fear) and several changes are noted in the body, for example: dilated pupils, increased heart rate and blood pressure, redirected blood from the skin, decreased digestion and dilated bronchioles. During activation adrenocorticotropic hormone is released and induces secretion of cortisol [9]. These changes can be used as possible indicators of pain and several of these changes have been shown in piglets during and after castration [2,10,11].

Stress, trauma, infection or inflammation also triggers the acute phase protein response, which is a part of the body's early defence. Serum amyloid A (SAA) is a major acute phase protein in pigs that can increase quickly and with large amplitude, and SAA level can therefore be used for defining the health and welfare status of pigs [12].

During castration, piglets without anaesthesia produce resistance movements with longer duration and higher intensity than piglets with anaesthesia [13]. After castration, behaviour alterations show that pain responses induced by castration persists over time; up to four to six days after castration according to some studies [5,14,15]. The pig production sector is searching for suitable methods that reduce pain induced by surgical castration, and alternatives to surgical castration. The method must be fast, cost effective, produce minimum stress and pain both during and after castration, and be safe for both the handler and the piglet. The method should also ensure a quick recovery to minimize the risk of the piglet being crushed by the sow. Currently there are essentially two alternatives that meet most of these requirements and which could be accepted in Swedish pig production. One method is immunocastration and the other involves the use of local anaesthesia and analgesia.

The objective of this study was to evaluate pain-related responses of male piglets castrated with/without local anaesthesia and with/without analgesia. A second purpose was to evaluate if herdsmen can effectively administer local anaesthesia by intratesticular injection. If the outcome of the study shows that herdsmen are able to effectively administer local anaesthesia this can lead to change of regulation, which will make it possible for herdsmen in Sweden to anaesthetise their piglets before castration. The use of anaesthetics in EU countries and Norway is currently restricted to veterinarians (Council Regulation No 2377/90). Herdsmen are allowed to administer analgesia after approved education according to Swedish regulation (SJVFS 2010:17, D9).

## Methods

The study has been approved by the Ethical Committee for Animal Experiments, Uppsala, Sweden (reference number C 164/9). All piglets in the study would have been subjected to castration as a routine procedure, regardless of the study.

### Herds, animals and management

The study was conducted between October 2009 and February 2010 in five piglet-producing herds in the south-central part of Sweden. The herds were satellite herds within a sow pool with Landrace x Yorkshire sows, and the sires of the piglets were Hampshire boars. Batch-wise production was applied and in each batch about 45 sows farrowed in individual farrowing pens. The pens had a concrete floor, with a slatted floor in the dunging area and a nest area for the piglets with a heat lamp. Cross-fostering was applied and these piglets were not discriminated in the study. All piglets, except for those in herd 1, received an iron injection on the day of castration. Piglets in herd 1 were given oral iron pasta shortly after birth. No piglets were subjected to teeth clipping or tail docking, which is not allowed according to Swedish regulation.

Castration was performed on 1-7 days old piglets and the majority of the piglets were 3-4 days old when castrated. In all herds except for herd 2, the piglets were fixated in a restraining device and castration was carried out using a scalpel. The scalpel was used to make the initial incisions after which the testicles were severed by cutting the spermatic cords. In herd 2, the piglets were restrained between the herdsmen's legs or under their arm and castration was performed using an emasculator. The emasculator was used to make the initial incisions after which the testicles were removed by cutting the spermatic cords.

The study comprised 557 male piglets, randomly selected from five herds. In these herds, 30, 25, 30, 26 and 30 experimental litters were included.

### Experimental design

Four male piglets in each of 141 litters were randomly assigned to one of four treatments: castration without local anaesthesia or analgesia (C, controls), castration with analgesia (M, meloxicam), castration with local anaesthesia (L, lidocaine), or castration with both local anaesthesia and analgesia (LM). All four treatments were represented in each litter. Six litters were made up of only three male piglets, and one litter of only two. Seven litters were therefore incomplete and the total number of male piglets was lower than optimal 564.

Before the study started, the herdsmen received instructions from a veterinarian on how to inject local anaesthesia and analgesia.

Two technicians, who were not blind to the treatments due to practical reasons, performed all measurements. The measurements were split between the two technicians with each technician performing the same measurements in all herds.

### Drugs

For local anaesthesia, lidocaine 10 mg/ml with epinephrine 5 μg/ml (Xylocain®, AstraZeneca, Södertälje, Sweden) was used (treatments L and LM). A total of 0.5 ml was injected in each testicle. While most of it was administered into the testicle, a small amount was injected subcutaneously into the scrotum when pulling the needle out. The action time of lidocaine is approximately one hour [16]. Castration was performed three minutes to 30 minutes after injection of lidocaine.

For analgesia, the nonsteroidal anti-inflammatory drug (NSAID) meloxicam 5 mg/ml (Metacam®, Boehringer Ingelheim Vetmedica, Malmö, Sweden) was used (treatments M and LM). A dose of 0.2 ml was injected intramuscularly behind the piglet's ear immediately after the castration.

### Measurements

On the castration day, the four males in each litter subjected to the study were weighed and marked with spray colour on their back. Each treatment was represented by a different colour. Lidocaine was injected (by herdsmen) to the piglets subjected to treatments L and LM. The castration was performed in random order within each litter. During castration, piglet vocalisation was measured and resistance movements were judged. Vocal response was measured with a decibel meter (Mini Sound Level Meters) measuring dB(A) and the call with the highest intensity level during the castration was recorded. The decibel meter was held as close to the snout as possible without touching it. Resistance movements were judged on a visual analogue scale (VAS) [17] where a mark closer to the left end of the line corresponds to "low intense" movements and a mark closer to the right end corresponds to "high intense" movements. The piglets were ranked on a 1-4 scale (most-least) within each litter according to the intensity and duration of their resistance movements. After castration, piglets subjected to treatments M and LM were given meloxicam (by herdsmen).

After castration, piglet behaviour was observed through instantaneous observations every ten minute, during 70 minutes, resulting in seven observations per piglet. Each technician studied ten litters per herd, and together a total of 398 piglets from 100 litters. A detailed ethogram with 23 variables (Table 1) with behaviours suggested by Wemelsfelder and van Putten [5], Hay et al. [14] and Llamas Moya et al. [15] was used. The behaviours were classified into five groups: body position, non-specific behaviour, pain-related behaviour, social cohesion and location. The pigs were studied from the front of the pen.

**Table 1:** Description of the behaviours of piglets [following 5,14,15]

**1. Body position**	
Standing	Body weight supported by four legs
Kneeling	Body weight supported by front carpal joints and hind legs
Dog-sitting	Body weight supported by hindquarters and front legs
Ventral lying, belly	Body weight supported by belly
Lateral lying, side	Body weight supported by side
	
**2. Non-specific behaviour**	
Walking/running	Moving walking, trotting or galloping
By udder	Activity by the udder: suckling, massaging udder or looking for a teat
Nosing/chewing/licking	Nosing/chewing or licking material or the littermates/mother
Playing	Head shaking, springing (sudden jumping or leaping) or running. Can involve partners (gentle nudging or pushing, mounting, chasing, etc.)
Sleeping	Eyes closed while lying
Awake inactive	Eyes open doing nothing
	
**3. Pain-related behaviour**	
Huddled up	Lying with at least three legs tucked under the body
Spasms	Quick sudden involuntary contractions of the muscles under the skin
Rump-scratching	Scratching the rump by rubbing it against the floor, pen walls or mother
Stiffness	Lying with extended and tensed legs
Prostrated	Sitting or standing motionless, with head down, lower than shoulder level
Trembling	Shivering as with cold. The animal may be lying, sitting or standing
	
**4. Social cohesion**	
Isolated	Aside from other piglets, alone. A distance of at least ~40 cm separates the animal from the closest littermate
Desynchronised	Activity different from that of most (at least 75%) littermates (e.g. sleeps while most other littermates suckle)
	
**5. Location**	
Heat-lamp	Sitting, standing, lying under the heat lamp

The following morning behaviour was observed according to the same protocol as on the castration day. Subsequently, castration wounds of the experimental piglets were ranked on the basis of how swollen the wounds were using a 1-4 scale (most-least) within the litter.

Temperature was measured the following day using digital infrared thermometers. The thermometer for ear temperature measured with an accuracy of ± 0.1°C. Skin temperature was measured around the castration wounds, the thermometer had an accuracy of ± 0.2°C.

In each herd blood samples were collected from piglets in 15 litters, a total of 296 piglets. Plain vacutainer tubes were used for collecting ~2 ml blood per piglet. The samples were centrifuged at 2000 x *g *at 5°C for five minutes two to five hours after the collection. The plasma was stored into cryo tubes in -20°C until they were analysed for SAA with commercial solid phase sandwich immunoassay kit (Tridelta Ltd.) in accordance with the manufacturer's instructions. The detection limits were 15.6 - 2000 mg/l.

Finally, the piglets were marked with different-coloured ear tags depending on the treatment and the litter identity was written on the tag.

At three weeks of age, the piglets were weighed again and the ear tags were removed. Journals that the herdsmen had kept for registration of sickness treatments and mortality were collected.

### Statistical analysis

Statistical analysis was performed using SAS Software, version 9.2 (SAS Institute Inc., Cary, NC, USA). Normal distribution was checked using proc univariate. Vocalisation, ear and skin temperature, and weight gain were analysed using analysis of variance (proc mixed). The statistical model applied (Model 1) included the fixed effects of herd (5 classes, herd 1-5), treatment (4 classes, C, M, L and LM), the interaction between herd and treatment, and the random effect of litter nested within herd.

Rank data of resistance movements and swelling of wounds was analysed using proc npar1way. Kruskal-Wallis test was applied and pair-wise comparison between treatments was performed using Wilcoxon tests.

The 23 behaviour variables, and five constructed group variables (Table 1), which for each piglet was the sum of all seven 0/1-observations, were analysed using proc glimmix (Model 1, poisson distribution). The group variables were the sum, for each piglet and day, of observations with at least one behaviour variable occurring within a group. The two observation occasions (the castration day and the following day) were analysed separately.

The effect of lidocaine and meloxicam was tested by considering them two separate treatments: lidocaine administered (L+LM), lidocaine not administered (C+M), meloxicam administered (M+LM) and meloxicam not administered (C+L). The statistical model (Model 2) included the fixed effects of a herd (5 classes, herd 1-5), administration of lidocaine (2 classes, 0/1), administration of meloxicam (2 classes, 0/1), the interaction between administration of lidocaine and meloxicam, and the random effect of litter nested within herd.

The data on SAA concentration was transformed into a number of 0/1-variable. Each value was assigned a 1 when the SAA concentration exceeded a certain threshold value (50, 100, 200, 400 and 600 mg/l). The analysis was performed using proc glimmix (Model 2, binomial distribution).

Sickness treatment and piglet mortality were analysed using X^2^-test. Correlations were calculated using spearman rank correlation. P-values ≤ 0.05 were regarded as significant.

## Results

The results are presented for all four treatments. However, treatments C and M were identical during castration, i.e. the piglets were castrated without lidocaine. Treatments L and LM were also identical as these piglets were castrated with lidocaine. The following day, treatment C and L were relatively comparable because the piglets were not given meloxicam, while the piglets in treatments M and LM had been treated with meloxicam. The interaction between herd and treatment did not have any significant impact on the variables but was included in the model to demonstrate this.

### Vocalisation and resistance movements during surgical castration

**Figure 1:**
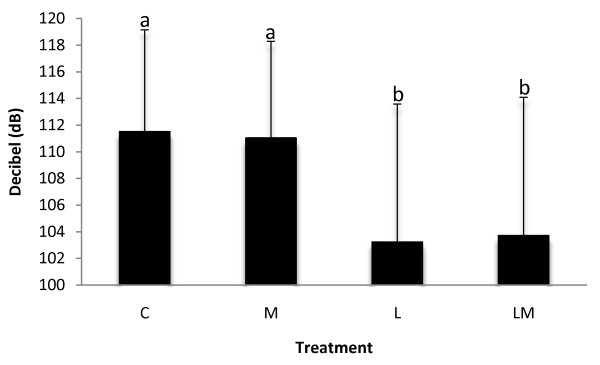
**Effect of treatment on call intensity**. Mean value and SD for call intensity (dB(A)) for the treatments C, M, L and LM. Piglets castrated with lidocaine (L and LM) produced calls with significantly (*p *< 0.001) lower intensity than piglets castrated without lidocaine (C and M). Means with different letters indicate significant differences (*p *< 0.05).

As shown in Figure 1, piglets castrated with lidocaine (L and LM) produced calls with a lower intensity level (*p *< 0.001) than piglets castrated without lidocaine (C and M). There were no significant differences between the two treatments with lidocaine (L and LM) or between the two treatments without lidocaine (C and M).

**Figure 2:**
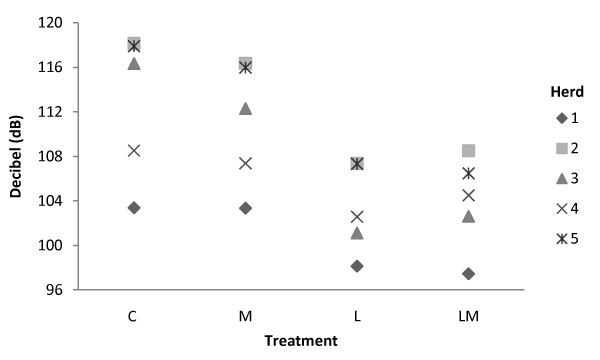
**Effect of treatment on call intensity in the five herds**. Mean value for call intensity (dB(A)) for the treatments C, M, L and LM, per herd. There were no significant interactions between treatment and herd.

Figure 2 shows the difference in call intensity between the herds. Significant interaction between treatment and herd was not found for call intensity.

**Figure 3:**
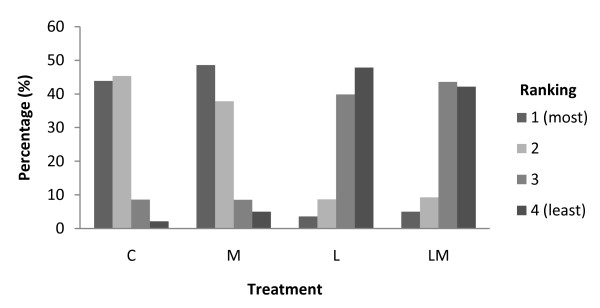
**Ranking of resistance movements during castration**. Piglets castrated with lidocaine (L and LM) showed significantly (*p *< 0.001) less resistance movements than piglets castrated without lidocaine (C and M).

Piglets castrated with lidocaine (L and LM) showed less resistance movements (*p *< 0.001) than piglets castrated without lidocaine (C and M), (Figure 3). No significant difference in resistance movements was found between the two treatments with lidocaine (L and LM) and the two treatments without lidocaine (C and M).

The correlation between call intensity and resistance movements was *r *= -0,38 (*p *< 0.001).

### Physiological responses to surgical castration

**Figure 4:**
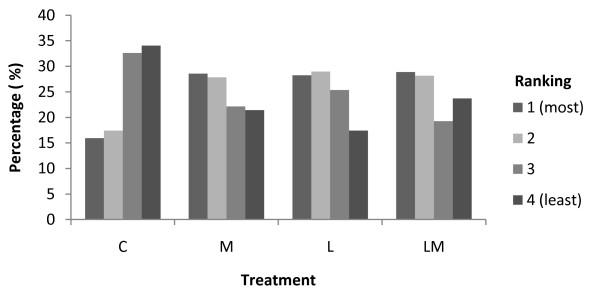
**Ranking of castration wounds swelling the day after castration**. Controls (C) had significantly less swollen wounds compared with treatments M, L and LM (*p *< 0.001).

Controls had less swollen castration wounds compared to the other three treatment groups (*p *< 0.001), (Figure 4). There was no significant difference between treatments M, L and LM.

Ear temperature was significantly higher (*p *< 0.01) for controls compared to piglets given lidocaine (L and LM), (Table 2). No significant differences were found for skin temperature. Within treatment, SD for temperature (both ear and skin) were higher for L (1.1°C) compared with the other three treatments (0.9°C).

**Table 2:** Mean value and SD for ear and skin temperature for the different treatments the day after castration

	Treatment			
	
	C	M	L	LM
Ear temperature				
Mean (°C)	38.5^a^	38.4^ab^	38.2^b^	38.3^b^
SD	0.9	0.9	1.1	0.9
Skin temperature				
Mean (°C)	35.8	35.8	35.9	35.8
SD	0.8	0.9	1.1	0.8

Ear temperature was significantly higher (*p *< 0.01) for controls (C) compared to piglets given lidocaine during castration (L and LM). Means with different letters indicate significant differences (*p *< 0.05)

For the SAA 0/1-variables, pair-wise comparisons between the four treatments did not show significant differences (Table 3). However, significant effects for the threshold value 200 and 400 mg/l (*p *= 0.005; *p *= 0.05) were found when treatments C+L (no meloxicam) were compared with treatments M+LM (meloxicam). For the threshold value 600 mg/l there was a tendency for a lower proportion of piglets given meloxicam (*p *= 0.06, n.s.).

**Table 3:** Proportion piglets with SAA concentrations over stated threshold value, per treatment

	Treatment							
	
Threshold value (mg/l)	C	M	L	LM	C+M	L+LM	C+L	M+LM
50	51	48	54	57	50	56	52	52
100	35	32	45	37	34	40	40	34
200	25	17	36	16	21	25	30	16 **
400	20	11	15	8	15	11	17	9 *
600	15	6	8	5	9	6	11	5 ^(*)^

The proportion of piglets with SAA threshold value over 200, 400 and 600 mg/l was higher for piglets not given meloxicam (C+L) compared to piglets given meloxicam (M+LM) ( ** *p *= 0.005; * *p *= 0.05; (*) *p *= 0.06)

Herd 1 had a lower proportion of piglets with high SAA concentrations. The percentage of piglets not given meloxicam that were above threshold value 200 mg/l was 13% in herd 1 compared to 37% in the other herds. For piglets given meloxicam, the percentage of piglets above 200 mg/l was 7% for herd 1 and 21% for the other herds. In herd 1, none of the piglets given meloxicam had SAA concentrations over 400 mg/l. In the other herds, at least one piglet given meloxicam had SAA concentrations over the thresholds 400 and 600 mg/l.

A total of 63 piglets (11%) were treated for health problems between the castration day and three weeks of age. The piglets were equally distributed over the treatments (C:17, M:11, L:17 and LM:18). During the same period, 26 piglets (5%) died but no significant effect of treatment on mortality was found (C:6, M:6, L:4 and LM:10).

The mean weight on the castration day was 2.2 kg (SD = 0.5 kg) for all treatments. There was no significant difference between treatments in weight gain (kg) between the castration day and three weeks of age.

### Behavioural responses to surgical castration

No significant treatment effects in behaviour were found related to any of the 23 behaviour variables when these were analysed separately (using Model 1). When the variables were categorised into the five groups (Table 1), a significant difference was found between treatments C and LM for the group pain-related behaviour (huddled up, spasms, rump-scratching, stiffness and prostrated), on the day after castration (*p *= 0.04), (Table 4).

**Table 4:** Percentage of displayed behaviours the castration day (day 0) and the following day (day 1) for each treatment

	Day	C	M	L	LM
Body position	0	48.3	44.8	45.2	44.7
	1	41.9	39.3	40.7	38.9
Non-specific behaviour	0	71.1	75.0	74.6	75.3
	1	74.7	77.5	73.9	74.9
Pain-related behaviour	0	6.0	4.6	6.5	4.7
	1	5.8^a^	4.3^ab^	5.9^ab^	3.6^b^
Social cohesion	0	3.5	2.7	2.5	3.7
	1	2.2	2.1	2.3	1.6
Location-heat lamp	0	55.9	52.3	52.9	52.3
	1	51.2	48.3	50.4	52.2

Significance was found between treatments C and LM for pain-related behaviour (*p *= 0.04) the day after castration. Means with different letters indicate significant differences (*p *< 0.05)

The effect of lidocaine and meloxicam was tested by considering them as two separate treatments: lidocaine administered (L+LM), lidocaine not administered (C+M), meloxicam administered (M+LM), meloxicam not administered (C+L), (using Model 2), (Table 5). The comparisons showed that piglets given meloxicam (M+LM) displayed less pain-related behaviour than piglets not given meloxicam (C+L) on both the castration day (*p *= 0.06, n.s.) and the following day (*p *= 0.02). No significant difference was found between treatments L+LM and C+M for pain-related behaviour. No significant differences were found in the other four groups of behaviour variables.

**Table 5:** Percentage of displayed behaviours the castration day (day 0) and the following (day 1) when lidocaine respectively meloxicam was administered or not

	Day	L+LM	C+M	M+LM	C+L
Body position	0	44.7	48.3	44.8	45.2
	1	38.9	41.9	39.3	40.7
Non-specific behaviour	0	75.3	71.1	75.0	74.6
	1	74.9	74.7	77.6	73.9
Pain-related behaviour	0	4.7	6.1	4.6^(a)^	6.5^(b)^
	1	3.6	5.8	4.3^a^	6.0^b^
Social cohesion	0	3.7	3.5	2.7	2.4
	1	1.6	2.2	2.1	2.3
Location-heat lamp	0	52.3	55.9	52.6	52.9
	1	52.2	51.2	48.3	50.4

Piglets given meloxicam (M+LM) showed less pain-related behaviour than piglets not given meloxicam (C+L) on both the castration day (day 0, *p *= 0.06, n.s.) and the following day (day 1, *p *= 0.04). Means with different letters indicate significant difference (*p *< 0.05).

## Discussion

### The method

The present study has, in line with several other studies, shown that castration without anaesthesia causes severe pain [2-4,13]. This pain persists for several days [5,14,15] and can cause delayed recovery, reduced feed-and water intake, reduced immune capacity and impaired welfare [18]. Traumatic experiences of pain, such as that experienced during castration, can also lead to hypersensitivity [19 cited by 10] and may result in increased stress when piglets associate handling with acute pain [11].

The outcomes of the study show that the herdsmen in the study were able to administer local anaesthesia effectively into the testicles and scrotum so that an adequate anaesthesia was achieved. This method is a possible way forward for improved welfare for male piglets in Swedish pig production. The herdsmen must however be instructed by a veterinarian before being allowed to administer local anaesthesia. Precision of the injection and the waiting time after injection affects the efficiency of the anaesthetics [16] and have to be focused in the training.

Possible methods that do not involve surgical castration are immunocastration and raising of entire males. However, raising of entire males does also affect the animal welfare negatively because of aggressive behaviour and mounting leading to e.g. increased leg problems [20]. To slaughter entire males before sexual maturity is not economically sustainable in Sweden. Castration under CO_2 _-gas anaesthesia is not a suitable method according to Swedish animal welfare legislation. CO_2 _-anaesthesia induces a high level of stress in the early induction phase, before surgical anaesthetic depth is reached [21]. In addition, use of anaesthetic gases is strictly regulated by the Swedish work environment act (ASS 2001:7) and is not suitable for field use [22].

Lidocaine was chosen for local anaesthesia as it has been used in several studies concerning castration and beneficial effects have been identified [2-4,16,23]. Lidocaine has a rapid onset and low toxicity [24]. The effect of the anaesthesia is prolonged by epinephrine and the risk for systemic reactions, e.g. fever, apathy and inappetence, decreases [24]. Ranheim et al. [16] showed that 40 min after injection the lidocaine concentration in the cords was severely decreased and this may have affected the result of the few piglets with long interval (up to 30 min) between lidocaine injection and castration.

In the present study all piglets received 0.2 ml meloxicam regardless of weight. This can have implications for low and heavy weight piglets and might have affected the result. In practice it will probably not be possible to give the exact dose of meloxicam given the weight. Boehringer Ingelheim recommends a dose of 0.2 ml for a piglet weighing 2.5 kg [25]. To be able to evaluate the effect of the drugs during the castration, meloxicam was given after the castration. In practice it would be possible to give meloxicam in connection with the injection of the local anaesthesia. There was a variation in time between the meloxicam administration and the start of the behaviour observations and this might have affected the results of the behaviour study on the castration day.

### Vocalisation and resistance movements during surgical castration

In agreement with other studies on piglet vocalisation during castration [3,4], the present study shows that piglets castrated with lidocaine produced calls with a lower intensity than piglets castrated without lidocaine. Marx et al. [4] have shown that calls produced by piglets castrated with lidocaine are similar to those produced by sham-castrated piglets.

Marx et al. [4] have suggested that a parameter that describes a single moment in the call, e.g. peak level, is more representative than parameters that describe a mean level. Therefore, in this study the call with the highest intensity during castration was recorded. It is assumed that this call was produced during the pulling and severing of the spermatic cords, which Taylor and Weary [8] have identified as the most painful moment during castration.

A difference in call intensity between the herds may be explained by the different castration techniques used by the herdsmen. The calls with the highest intensity were recorded in herd 2 where the herdsmen used an emasculator for castration. This can be interpreted that castration with an emasculator causes more pain, but it is more likely because the calls in herd 2 were not suppressed by the restraining device.

However, restraining method has been seen to not influence the pain responses during castration [6]. Taylor and Weary [8] state that it might be the pulling of the spermatic cords more than the severing that is painful. Traction upon the testes is likely to be felt along the spermatic cords and into the inguinal canal. If the testicles and spermatic cords are pulled a long distance before severing, this is likely to cause pain that may not be prevented by local anaesthesia in the testicles. Even after intratesticular injection of lidocaine the piglets still responded with some vocalisation and resistance movements during the castration procedure. Ranheim et al. [16] showed by means of autoradiograms that radiolabelled lidocaine injected into a piglet testicle was evident in both testicle and spermatic cord three minutes after injection. However, the concentration in the cremaster muscle was low ten minutes after injection. As the cremaster muscle is cut off during castration this can explain why piglets show some pain-related behaviour despite receiving lidocaine.

The study showed a correlation between dB-level and resistance movements. High dB-levels were associated with intensive resistance movements. In this study, as well in studies by Leidig et al. [13] and Horn et al. [23], local anaesthesia (procaine and lidocaine) reduced resistance movements during castration.

### Physiological responses to surgical castration

The study showed that controls had less swollen wounds compared to piglets treated with lidocaine or meloxicam. Small bleedings can occur as a result of the injection of local anaesthetics, which can contribute to an increased swelling (personal communication, Nyman, 2011). Why the swellings also were increased for piglets treated with meloxicam cannot be explained. The result is similar to Kluivers-Poodt et al. [3] where thickening of the scrotum was found on the fourth day after castration in several piglets treated with lidocaine and/or meloxicam.

Rectal temperature is the best indicator of adequate body temperature, but measuring temperature in the ear can also provide reliable estimates of body temperature and was used since it is a very fast method. Body temperature is nearly constant but fever occurs because of infections, or in some cases extensive tissue damage. Skin temperature is on the other hand more influenced by the environment and can therefore vary considerably. During activation of the sympathetic nervous system the blood is redirected from the skin to essential organs, which leads to a lowering of the skin temperature [9], and measurements can give information on the shock reaction [3].

In present study measurements of ear temperature showed that controls had higher temperature than piglets given lidocaine, why cannot be explained. Hypothetically, piglets given meloxicam should have lower ear temperature than piglets not given meloxicam because of the NSAIDs antipyretic effects [26]. No differences between treatments were found in terms of skin temperature and this is probably because the measurements were performed the day after castration, when the skin temperature had returned to normal.

The SAA concentration in the blood is normally very low (bordering on the unmeasurable) [12] but can increase hundredfold after stress, trauma, infection or inflammation as a consequence of increased levels of pro-inflammatory cytokines [27]. The concentration is the highest two to three days after a trauma and return to normal levels after seven to ten days [12,28]. The SAA concentration can act as an general marker of inflammation and has been seen to reflect the intensity of stress, trauma and inflammation [28-30]. The results from the present study show that piglets that were given meloxicam had lower SAA concentrations on the day after castration. The percentage of piglets with high SAA concentrations (> 200 mg/l) was halved when meloxicam was administered. The enzyme cyclooxygenase (COX) is a prerequisite for the creation of prostaglandins, the proteins that create pain-mediating substances [26]. NSAIDs act anti-inflammatory trough inhibition of COX [26] and the result of the present study can be seen as an indirect measure of the anti-inflammatory effect of the meloxicam. The action time for lidocaine is limited to approximately one hour and is not likely to affect the postoperative inflammation [28].

SAA analysis showed a deviating pattern for piglets given meloxicam in herd 1: the proportion of piglets with high SAA concentrations was much lower compared to other herds. Piglets in herd 1 were given oral iron pasta after birth instead of an iron injection on the castration day. Injection of iron is an unnatural way for piglets to receive iron because there is no regulation system for exudation of iron trough the liver or kidneys. In nature, iron enters the body exclusively through the diet. The iron balance is regulated by the rate of absorption from the small intestine and the risk for extreme concentrations is therefore lower when iron is given orally compared with injection [31]. Addition of iron salts in connection with injection of NSAID might increase the irritating effect on gastrointestinal mucous [32] and that might have caused the higher SAA concentrations in the other herds.

The weight gain did not differ between the treatment groups, which is in accordance with other studies [3,5,14,33].

### Behavioural responses

In the present study, piglets showed specific pain-related behaviour induced by castration which also has been seen in other studies [3,5,14,15,33]. The piglets given meloxicam in the present study showed less pain-related behaviours than piglets not given meloxicam. Similary, Keita et al. [33] have found an effect of meloxicam on pain relief two and four hours after castration. Kluivers-Poodt et al. [3] have also seen that piglets castrated with or without lidocaine showed more pain-related behaviours than sham-castrated piglets. However, less pain-related behaviours were displayed if the piglets with lidocaine were also given meloxicam.

Differences in piglets' non-specific behaviour between the treatments were not shown in this study. Other studies have shown that castrated piglets become more isolated after castration [14,15] and that the time spent by the udder (both more and less) differs between castrated and sham-castrated piglets [7,14,15,34]. However, in present study, no sham-castrated were included, but only castrated piglets. A lack of differences in non-specific behaviour can also be explained by the fact that all treatments were present in the same litter, which may have caused, as suggested by Kluivers-Poodt et al. [3], the piglets to influence each other's social behaviour.

## Conclusions

This study concludes that lidocaine injected intratesticularly reduced pain responses during castration and that meloxicam reduced the pain-related behaviours after castration. It is therefore recommended that both local anaesthesia and analgesia should be given to piglets to reduce pain induced by castration. The study also concludes that the herdsmen in the study, after training, were able to inject local anaesthesia effectively. However, the method requires handling of the piglets on two separate occasions, which contribute to stress.

## Competing interests

The author declares that they have no competing interests.

## Authors' contributions

MH participated in developing the design of the study, performing the field study, analysing data and drafting the manuscript. NL applied for funding of the study, planned the design of the study, helped analyse data and helped to draft the manuscript. GJ and GN participated with veterinarian expertise to the design of the study and education of the herdsmen. All authors have read and approved the final manuscript.
